# Tailored Functionalized Magnetic Nanoparticles to Target Breast Cancer Cells Including Cancer Stem-Like Cells

**DOI:** 10.3390/cancers12061397

**Published:** 2020-05-29

**Authors:** Ana Lazaro-Carrillo, Macarena Calero, Antonio Aires, Aitziber L. Cortajarena, Bruno M. Simões, Alfonso Latorre, Álvaro Somoza, Robert B. Clarke, Rodolfo Miranda, Angeles Villanueva

**Affiliations:** 1 Departamento de Biología, Universidad Autónoma de Madrid, Darwin 2, 28049 Madrid, Spain; ana.lazaro@uam.es (A.L.-C.); macarena.calero@imdea.org (M.C.); 2 Instituto Madrileño de Estudios Avanzados en Nanociencia (IMDEA Nanociencia), Faraday 9, 28049 Madrid, Spain; aaires@cicbiomagune.es (A.A.); alcortajarena@cicbiomagune.es (A.L.C.); alfonso.latorre@imdea.org (A.L.); alvaro.somoza@imdea.org (Á.S.); rodolfo.miranda@imdea.org (R.M.); 3 Departamento Química Física, Universidad Complutense de Madrid, Avda. Séneca 2, 28040 Madrid, Spain; 4 Center for Cooperative Research in Biomaterials (CIC biomaGUNE), Basque Research and Technology Alliance (BRTA), Paseo de Miramón 182, 20014 Donostia San Sebastián, Spain; 5 Ikerbasque, Basque Foundation for Science, 48013 Bilbao, Spain; 6 Breast Cancer Now Research Unit, Division of Cancer Sciences, University of Manchester, Manchester Cancer Research Centre, 555 Wilmslow Road, Manchester M20 4GJ, UK; bruno.simoes@manchester.ac.uk (B.M.S.); robert.clarke@manchester.ac.uk (R.B.C.); 7 Departamento de Física de la Materia Condensada, Universidad Autónoma Madrid, Francisco Tomás y Valiente 7, 28049 Madrid, Spain

**Keywords:** magnetic iron oxide nanoparticles, doxorubicin, mitotic catastrophe, senescence, apoptosis, cancer stem-like cells

## Abstract

Nanotechnology-based approaches hold substantial potential to avoid chemoresistance and minimize side effects. In this work, we have used biocompatible iron oxide magnetic nanoparticles (MNPs) called MF66 and functionalized with the antineoplastic drug doxorubicin (DOX) against MDA-MB-231 cells. Electrostatically functionalized MNPs showed effective uptake and DOX linked to MNPs was more efficiently retained inside the cells than free DOX, leading to cell inactivation by mitotic catastrophe, senescence and apoptosis. Both effects, uptake and cytotoxicity, were demonstrated by different assays and videomicroscopy techniques. Likewise, covalently functionalized MNPs using three different linkers—disulfide (DOX-S-S-Pyr, called MF66-S-S-DOX), imine (DOX-I-Mal, called MF66-I-DOX) or both (DOX-I-S-S-Pyr, called MF66-S-S-I-DOX)—were also analysed. The highest cell death was detected using a linker sensitive to both pH and reducing environment (DOX-I-S-S-Pyr). The greatest success of this study was to detect also their activity against breast cancer stem-like cells (CSC) from MDA-MB-231 and primary breast cancer cells derived from a patient with a similar genetic profile (triple-negative breast cancer). In summary, these nanoformulations are promising tools as therapeutic agent vehicles, due to their ability to produce efficient internalization, drug delivery, and cancer cell inactivation, even in cancer stem-like cells (CSCs) from patients.

## 1. Introduction

Approximately 50% of human cancer treatments are based on chemotherapy, being one of the most common strategies, mainly in metastatic disease. However, this method causes many side effects as well as multidrug resistance (MDR), one of the most severe problems during the treatment of oncological patients [1]. In this way, chemotherapy is usually employed to reduce the tumour size or when metastasis is produced and other strategies are not possible [2,3]. Generally, MDR reflects an overexpression of ATP-binding cassette (ABC) transporters on the plasma membrane, which are capable of causing an efflux of several anticancer drugs, such as doxorubicin, epirubicin, and paclitaxel [4,5].

Recently, nanotechnology-based formulations, or nanomedicine, have become a promising strategy for cancer treatment. This approach not only provides a new opportunity to overcome MDR, avoiding drug efflux, but also increases its retention time [6,7]. Magnetic nanoparticles (MNPs), which can be targeted to the tumour site with a magnetic field, gained importance in cancer therapy in recent years. This class of material includes metallic, bimetallic, and superparamagnetic iron oxide nanoparticles, which have a reactive surface that can be readily modified with biocompatible coatings and loaded with therapeutic agents [8,9]. The use of non-covalent functionalized magnetic nanoparticles with drugs to improve cancer therapy has been extensively explored to maximize the therapeutic activity of the drugs while minimizing their side effects [10,11,12]. However, there are still some drawbacks, such as the poor control of the release of the immobilized drug. Therefore, we propose the covalent functionalization of magnetic nanoparticles with drugs through stimuli-responsive linkers as a suitable way to tackle the controlled release. There is a growing interest in developing nanocarriers that can target disease sites in a specific manner and perform a gradual slow release, avoiding rapid efflux of chemotherapeutic drugs. Ideally, only cancer cells should be targeted by drug-functionalized magnetic nanoparticles, in which drugs are inactive until they are released inside the cells. In this regard, different linkers sensitive to intracellular triggering stimuli such as pH [13], reducing environment [14] and the presence of some enzymes [15], or external stimuli such as temperature [16] have been employed to attach and release drugs from magnetic nanoparticles in a controlled manner.

Regarding the specific targeting of tumours, recent research suggested that cancer originates from a small fraction of tumour initiating cells with self-renewal capability, unlimited propagation and multipotent differentiation. This small population of tumour cells has been called cancer stem-like cells (CSCs) [17,18]. Moreover, CSCs in tumours evade the anticancer effects of standard chemotherapy, emerging as an underestimated biological barrier to the success of systemic chemotherapy, inducing resistance to therapy and thus the subsequent tumour recurrence [19].

In this work, iron oxide magnetic nanoparticles were loaded with doxorubicin (DOX), an anticancer agent frequently used to treat different cancers [20]. Electrostatic and covalent strategies were employed to obtain DOX-loaded nanocarriers. The nanocarriers consist of a magnetic iron oxide nanoparticle (MNPs) with a coating of dimercaptosuccinic acid (DMSA) easily modifiable with different drug molecules. We applied different functionalization strategies to make a systematic comparative study to define the nanodelivery strategies that show advantageous therapeutic properties. Covalent delivery systems were designed by using three different linkers: disulfide (DOX-S-S-Pyr), imine (DOX-I-Mal), or both (DOX-I-S-S-Pyr). The purpose was to analyse which system was more effective in eliminating breast tumour cells, including breast cancer stem-like cells. Our results indicate that double-sensitive nanoparticles MF66-S-S-I-DOX have many of the properties that an ideal nanocarrier should have as stability, biocompatibility, sufficient loading capacity and ability to maintain its cytotoxic activity after their internalization by tumour cells, including against cancer stem cells.

## 2. Results

### 2.1. Functionalization of MF66

Firstly, biocompatible magnetic nanoparticles (MNPs) coated with dimercaptosuccinic acid (DMSA) called MF66 were successfully functionalized with DOX through electrostatic interactions. Secondly, DOX was covalently bound via: (1) disulfide bond (DOX-S-S-Pyr), a reducing environment sensitive linker; (2) imine bond (DOX-I-Mal), a pH-sensitive linker; (3) or both disulfide and imine bonds (DOX-I-S-S-Pyr) (see the Supporting Information for the detailed functionalization process and full physico-chemical characterization of resulting DOX-functionalized MNPs).

### 2.2. Drugs Release Studies

#### 2.2.1. Drug Release Studies from Electrostatic Functionalized MF66

**Figure 1:**
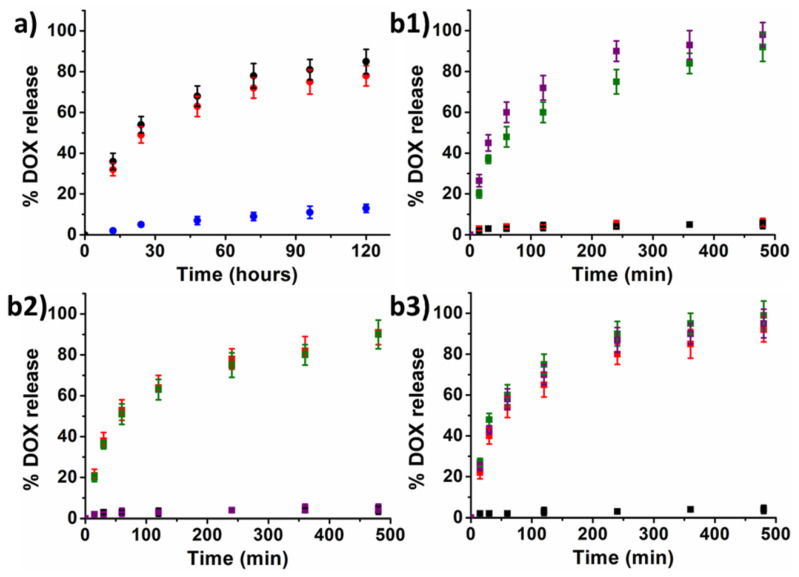
Release kinetics of the different formulations. (**a**) Release kinetics of the electrostatically immobilized doxorubicin (DOX) (circles) were studied by dispersing MF66-DOX in water (blue), PBS buffer (pH 7.4) (black), or sodium acetate buffer (pH 4.7) (red). (**b**) Release kinetics of the covalently immobilized DOX (squares) were studied by dispersing MF66-S-S-DOX (**b1**), MF66-I-DOX (**b2**), or MF66-S-S-I-DOX (**b3**), in PBS buffer (pH 7.4) containing 1 μM (black) or 1 mM of 1,4-dithiothreitol (DTT) (purple), or sodium acetate buffer (pH 4.7) containing 1 μM (red) or 1 mM of DTT (green).

The release of the attached molecules from the electrostatic functionalized MF66 was monitored for 120 h in milliQ^®^ water, PBS buffer (150 mM NaCl, 10 mM phosphate pH 7.4, extracellular pH) and AcONa/AcOH buffer (150 mM NaCl, 10 mM sodium acetate pH 4.7, intracellular pH) at 37 °C (Figure 1a).

These results show that physiological salt concentration in both PBS and acetate buffer caused the continuous dissociation of the molecules of DOX from the electrostatic functionalized MF66 and that the release rate was independent on the pH.

#### 2.2.2. Drug Release Studies from Covalent Functionalized MF66-MNPs

The release of the DOX from the covalent functionalized MF66 was monitored during 8 h in PBS buffer (150 mM NaCl, 10 mM phosphate pH 7.4) and AcONa/AcOH buffer (150 mM NaCl, 10 mM sodium acetate pH 4.7) using intracellular reducing conditions (1 mM of 1,4-dithiothreitol (DTT)) and extracellular reducing conditions (1 μM of DTT) at 37 °C (Figure 1b1).

These results show that in the case of MF66 functionalized with DOX-S-S-Pyr, a reducing environment caused the rapid dissociation of the drugs from MNPs and that the release rate was strongly dependent on the reducing environment. In the case of MF66 functionalized with DOX-I-Mal, an acidic environment caused the rapid dissociation of the drugs from MNPs, the release rate being strongly dependent on the pH (Figure 1b2).

Finally, in the case of MF66 functionalized with DOX-I-S-S-Pyr, both a reducing environment and/or acidic environment caused the rapid dissociation of the drugs from MNPs. Therefore, the cargo from the described nanoparticles is only released under reducing conditions and or acidic conditions as those present in the cytoplasm and lysosomal environments and is stable under extracellular neutral and low reducing conditions (Figure 1b3).

### 2.3. Effects of Electrostatic Formulation in Cell Culture

#### 2.3.1. Cell Viability Reduction with Functionalized MF66 with DOX

**Figure 2:**
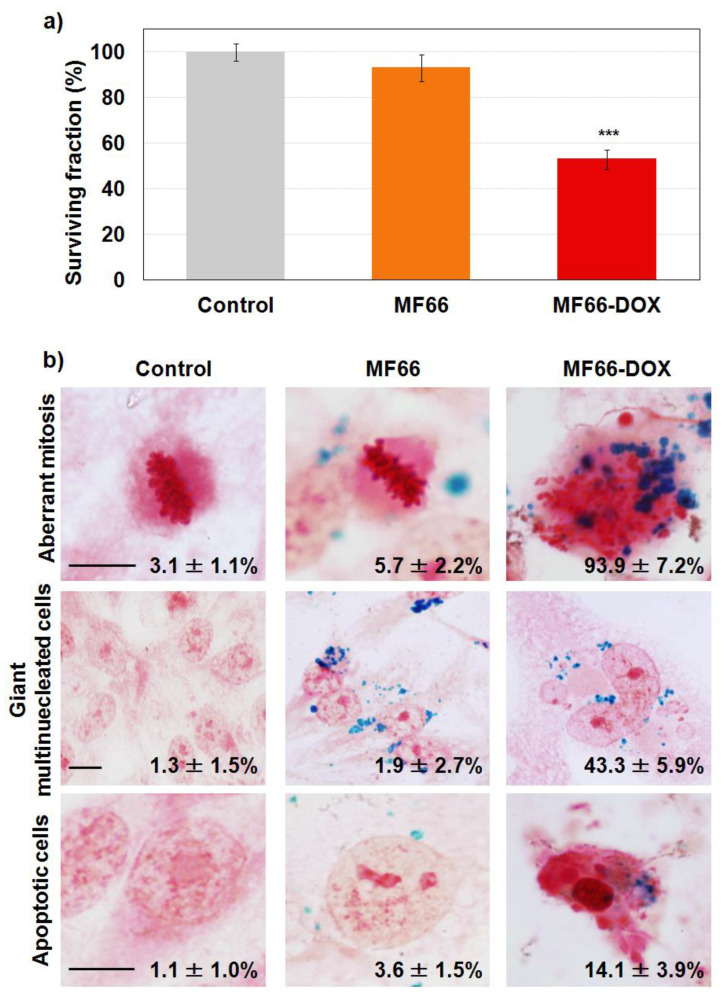
Evaluation of electrostatic nanoformulations in MDA-MB-231 cells 72 h after 24 h of incubation with the different treatments. (**a**) Cellular viability assessed after incubation with the different samples by AlamarBlue^®^ assay. (**b**) Efficient uptake and morphological alterations analysed by Prussian blue staining. Percentages included are the aberrant mitosis over the total number of mitosis and the number of giant multinucleated cells or apoptosis over the total number of cells. Scale bar: 10 µm.

As observed in Figure 2a, a decrease in cell viability was observed in samples treated with MF66-DOX (53.0% ± 4.3%). However, the cytotoxicity induced by free DOX was clearly higher (see Figure S1 of the Supplementary Material).

#### 2.3.2. Labelling Efficacy and Morphological Alterations

Staining with Prussian blue allowed us to visualize an efficient cell labelling with MF66. It also showed that incubation with MF66-DOX caused morphological alterations 72 h after treatment (24 h incubation with the different treatments). Cells incubated with bare MF66 showed no changes in cellular morphology (Figure 2b). However, in cells treated with MF66-DOX, a higher rate of mitosis appeared and most of the divisions were aberrant (multipolar, with altered mitotic spindles and misaligned chromosomes). In addition, the percentage of giant cells increased in cells incubated with DOX nanoformulation related to bare MF66 (43.3% ± 5.9% vs 1.9% ± 2.7%). Moreover, these cells had a single large nucleus or several nuclei, most of them with a smaller size or micronuclei. Finally, we also detected cells with apoptotic morphology (14.1% ± 3.9%).

#### 2.3.3. MF66 Formulation with DOX Triggered Cell Cycle Arrest, Apoptosis and Senescence

**Figure 3:**
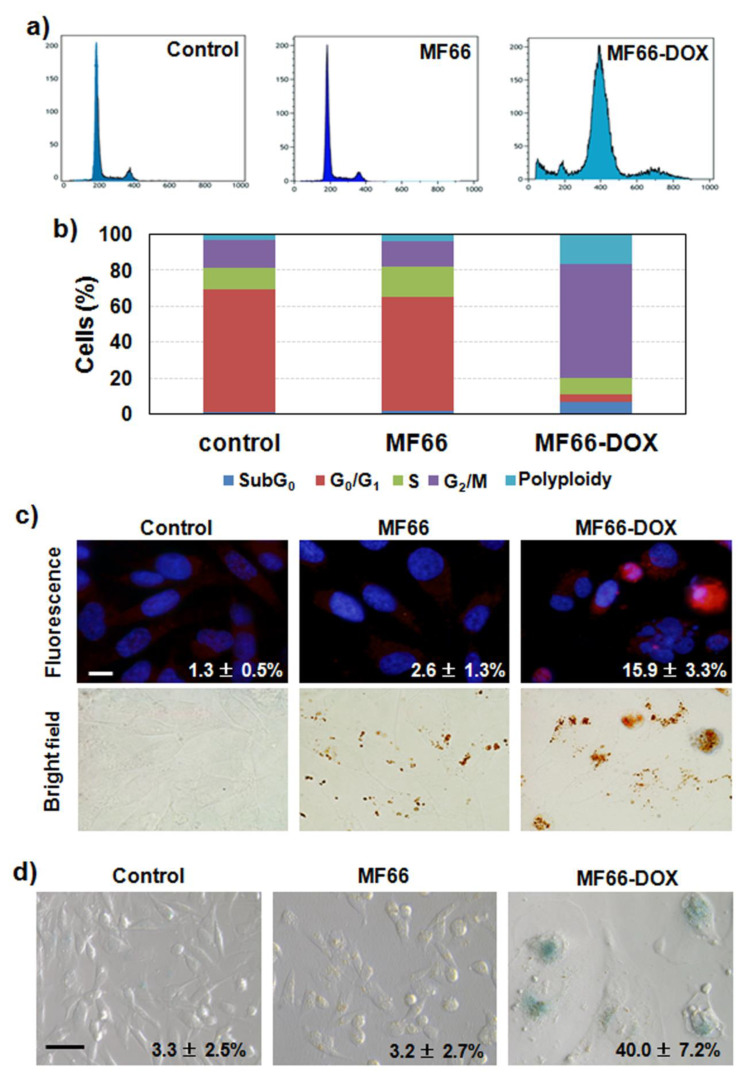
Cellular inactivation mechanisms analysed after incubation of MDA-MB-231 cells with electrostatic formulation. (**a**) Representative cell cycle histograms 72 h after 24 h of incubation with the different formulations. (**b**) Bar chart displaying percentages of cells in the different phases of cell cycle 72 h after 24 h treatments. (**c**) Apoptotic cells visualized by indirect immunofluorescence for cleaved caspase 3 (red) and DNA staining (blue) analysed 72 h after 24 h treatment. Scale bar: 10 µm. Percentages included are the apoptotic cells over the total number of cells. (**d**) Senescent cells analysed by senescence-associated β-galactosidase activity (blue) 96 h after treatment incubated for 24 h visualized in differential interference contrast (DIC) microscopy. Scale bar: 50 µm. Percentages included are the senescent cells over the total number of cells.

Flow cytometry by DNA staining with propidium iodide displayed a clear alteration of the cell cycle 72 h after the 24 h treatment with MF66-DOX (Figure 3a,b). In particular, a cell cycle arrest in the G2/M phase, as well as an increase in polyploidy rate, was observed. A peak in SubG0 phase was also detected, which could be related to the additional apoptotic cells detected with Prussian blue staining. This increase in apoptotic cell number was confirmed by caspase 3 activation assessed by immunofluorescence (Figure 3c). In addition, DNA counterstaining with Hoechst 33,258 displayed nuclear condensation and fragmentation. These are characteristic indications of apoptotic cell death.

Since the proliferation of surviving cells after DOX treatment was not observed, we proceeded to perform a test of cellular senescence based on the overexpression of senescence-associated β-galactosidase enzyme in the samples. The results show positive blue staining indicating senescence in almost half of the treated cells with MF66-DOX, with higher occurrence in the largest cells (Figure 3d).

All these morphological and biochemical changes were confirmed by time-lapse videomicroscopy. The full-length movies are available as Supplementary Material (Videos S1 to S3). At 48 h after treatment, cells were already larger, flatter and more frequently multinucleated (characteristic morphology of senescent cells) than control cells. In addition, movies of this sample display an apoptotic outbreak when approaching 72 h of post-incubation, and mitosis was longer than in the control or MF66 samples.

#### 2.3.4. Increase in DOX Retention with the Nanoformulation

**Figure 4:**
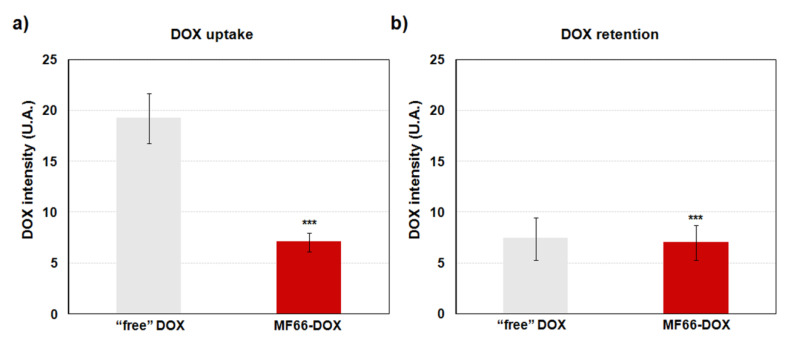
DOX efflux by MDA-MB-231 cells incubated with the drug dissolved in medium (“free” DOX) or linked to MF66. (**a**) Intracellular DOX level measured by flow cytometry after 24 h incubation. (**b**) DOX retention at 72 h after that 24 h incubation.

The flow cytometry results indicate that electrostatic formulation bound to DOX was internalized by MDA-MB-231 cells (Figure 4a), confirming the high internalization observed by Prussian blue staining. It should be taken into account that a higher uptake is reached by “free” DOX (drug non-loaded on MF66). However, it is important to highlight that while “free” DOX has a short retention time, the intracellular DOX level was significantly retained with MF66-DOX formulation at 72 h after that 24 h incubation (Figure 4b).

### 2.4. Effect of Covalent Formulations in Cell Culture

#### 2.4.1. Cell Viability Reduction with the Three Covalent MF66 Formulations with DOX

**Figure 5:**
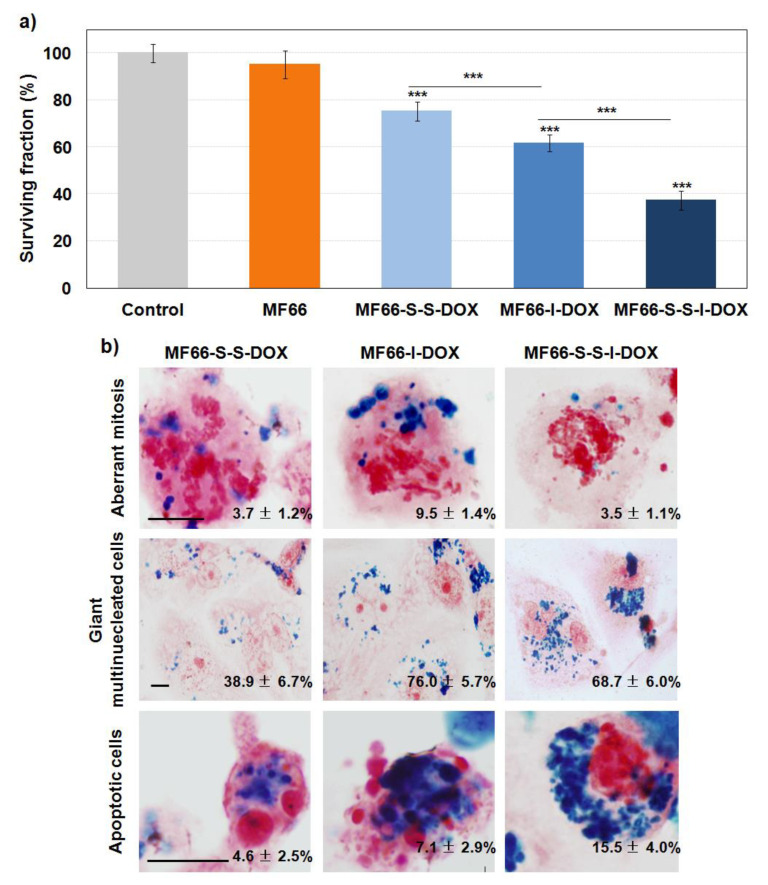
Effect of the different covalent formulations 72 h after the incubation for 24 h in MDA-MB-231 cells. (**a**) Cellular viability assessed by AlamarBlue^®^. (**b**) Efficient uptake and morphological effects analysed by Prussian blue staining. Scale bar: 10 µm. Percentages included are the mitosis, number of giant multinucleated cells or apoptosis over the total number of cells.

The AlamarBlue^®^ assay performed 72 h after incubation for 24 h with the different formulations demonstrated that cells treated with DOX-loaded formulations show a significant reduction in cell viability in all the cases, although it was higher with MF66-S-S-I-DOX (Figure 5a).

#### 2.4.2. DOX Covalent Formulations Have Similar Behaviour than the Electrostatic Formulation

Prussian blue staining confirmed similar results to those previously described for the electrostatic formulation with DOX. At 72 h after incubation for 24 h, this staining displayed a high internalization for the three MF66-DOX covalent formulations (Figure 5b). This result was confirmed by fluorescence microscopy. In the cells incubated with any of the three covalent formulations, the red signal from DOX was visualized intracellularly in the nucleus and cytoplasm, mainly attached to MNPs (Figure S1a).

A detailed study by differential interference contrast (DIC) microscopy allowed us to detect several morphological alterations in cells treated with MF66 covalently conjugated with DOX (Figure S1b). Prussian blue staining corroborated these results (Figure 5b). At this long post-incubation time, the mitotic index was tripled in MF66-I-DOX-treated cells in respect to control cells and aberrant mitosis was observed after incubation with this nanoformulation. Another relevant finding was the increase in multinucleated and giant cells. In addition, features of apoptosis (cell shrinkage, condensation, and fragmentation of nuclear chromatin) were also observed when the cells were treated with the three different samples, but mainly with MF66-S-S-I-DOX.

Taking into account that MF66-S-S-I-DOX, which had immobilized half the DOX compared to electrostatic formulation, led to the highest cytotoxic effect, mainly due to apoptosis, this covalent formulation was selected for activity evaluation on breast cancer stem cells in MDA-MB-231 cell line and primary metastatic patient-derived breast cancer cells.

#### 2.4.3. MF66-S-S-I-DOX Are Able to Target Breast Cancer Stem Cells

**Figure 6:**
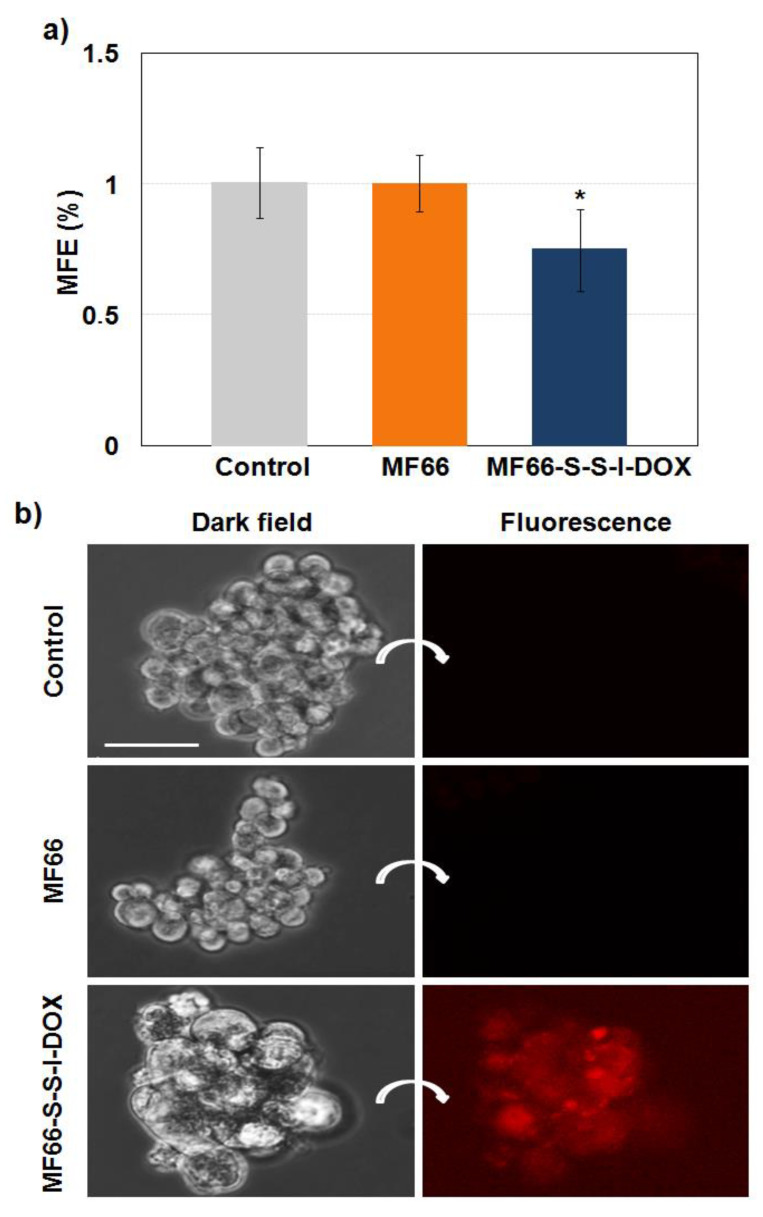
Mammospheres from MDA-MB-231 cells untreated (control) or treated with MF66 and MF66-S-S-I-DOX. (**a**) Quantification of mammosphere forming efficiency analysed 5 days after cell 24 h incubation with magnetic nanoparticles (MNPs). (**b**) Images of mammospheres in phase contrast and fluorescence microscopy 72 h after mammosphere formation assay. Scale bar: 50 μm.

The mammosphere colony assay was performed to quantify MDA-MB-231 cancer stem cell activity. Figure 6a displays significantly decreased mammosphere-forming efficiency 72 h after 24 h incubation with MF66-S-S-I-DOX compared to control cells. Images obtained by dark field and fluorescence microscopy revealed that mammospheres generated from treated cells with DOX formulation had a similar shape but with a lower number of cells, and most of the cells looked larger than control size ones (Figure 6b).

**Figure 7:**
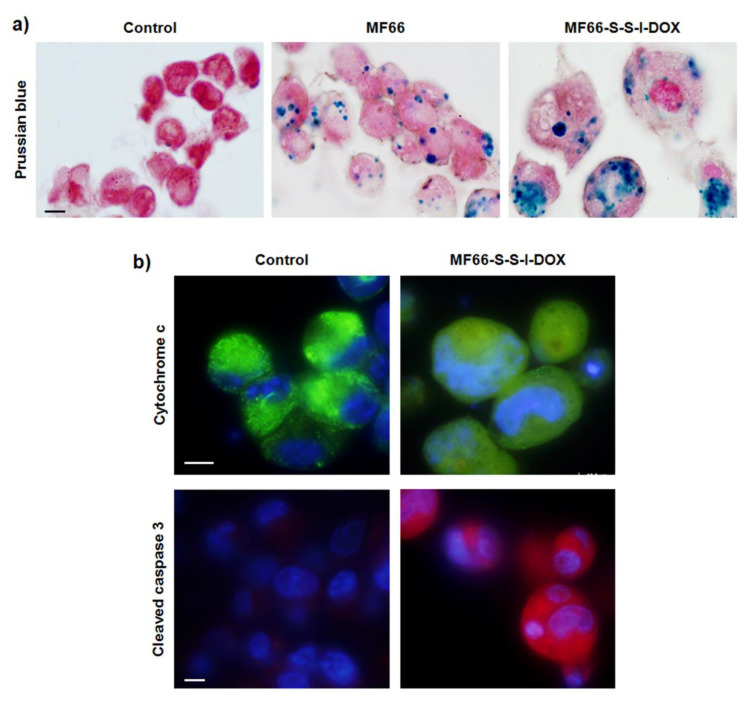
Morphological characterization and cell death mechanism triggered in mammospheres from MDA-MB-231 cells. (**a**) Prussian blue staining in control cells or treated with MF66 or MF66-S-S-I-DOX MNPs and visualized by bright field microscopy. Scale bar: 5 μm. (**b**) Immunofluorescence for cytochrome c (green) and DNA staining (blue) visualized by fluorescence microscopy. Scale bar: 5 μm. (**c**) Immunofluorescence for cleaved caspase 3 (red) and DNA staining (blue) visualized by fluorescence microscopy. Scale bar: 5 μm.

To characterize these mammospheres, paraffin-embedded sections were stained with Prussian blue, showing MNPs as blue spots inside cells (Figure 7a). Cells incubated with bare MNPs had similar morphologies to control cells (images not shown). However, mammosphere cells treated with MF66-S-S-I-DOX were larger and demonstrated nuclear condensation and fragmentation.

To confirm that several cells in mammospheres generated after treatment with DOX formulation had triggered apoptosis, immunostaining for cytochrome c and cleaved caspase-3 was performed in paraffin-embedded sections. Figure 7b shows cytochrome c as green spots in the control sections. However, cytochrome c appeared as a diffuse green signal in treated sections, indicating its translocation from mitochondria to the cytoplasm. Moreover, apoptosis in treated samples was confirmed by cleaved caspase-3 immunofluorescence, as illustrated in Figure 7b.

**Figure 8:**
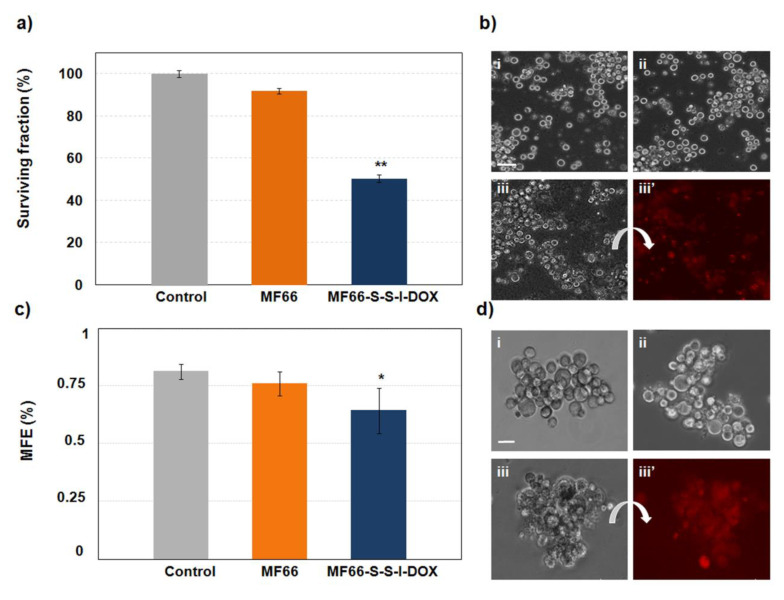
Analysis of MF66-S-S-I-DOX in primary metastatic patient-derived breast cancer cells. (**a**) Cellular viability assessed by trypan blue performed 24 h after the 24 h incubation. (**b**) Images of (i) control cells or incubated with (ii) bare MF66 or (iii, iii’) MF66-S-S-I-DOX in phase contrast and fluorescence microscopy, respectively, at the same post-incubation time. Scale bar: 50 μm. (**c**) Quantification of mammosphere forming efficiency analysed 7 days after cell incubation for 24 h with MNPs. (**d**) Images of mammospheres from (i) control cells or incubated with (ii) bare MF66 or (iii, iii’) MF66-S-S-I-DOX in phase contrast and fluorescence microscopy, respectively at the same post-incubation time. Scale bar: 25 μm.

Finally, we analysed the effect of this formulation in primary samples obtained from the pleural effusion from a patient with triple-negative breast cancer. Trypan blue staining showed a significant reduction in cell viability with MF66-S-S-I-DOX incubation for 24 h (Figure 8a). This was confirmed by direct observations of the cells by an inverted microscope, which, in addition, allowed us to detect fluorescence of the DOX (Figure 8b). In addition, the activity of cancer stem cells to form mammospheres was also significantly reduced (Figure 8c,d).

## 3. Discussion

MNPs have great relevance in the field of nano-oncology due to their multiple applications, including their use as mediators for different anti-tumour therapies (e.g., hyperthermia or carriers for chemotherapeutic drugs) [21]. For this purpose, two fundamental types of strategy have been used to design MNPs: non-covalent (electrostatic) and covalent interactions.

Cytotoxic analysis showed reduced viability of MF66 electrostatically functionalized with doxorubicin (DOX; MF66-DOX) in relation to bare MF66 in MDA-MB-231 cells 72 h after 24 h treatments. This result indicates the significance of long-term evaluation of nanoparticles’ effectiveness when they are functionalized, to understand the kinetics of drug release. In conventional cell culture studies, nanoparticles toxicity is not usually tested much longer than 24 h. It is important to note that MDA-MB-231 is a highly aggressive, invasive and poorly differentiated triple-negative (ER, PR and HER2 negative) breast cancer cell line and one of the most commonly used cell lines in cancer research laboratories [22].

It is also important to note that the cytotoxicity induced by free DOX was clearly higher compared to MF66-DOX (see Figure S1 of Supplementary Material). In relation to this fact, similar results have been described for other magnetic nanoparticles [23,24]. An adequate explanation for the differences observed in the cytotoxic effects of DOX and MF66-DOX could be their different transport mechanisms. Free DOX enters the cells very rapidly by simple passive diffusion and reaches the nucleus easily. In the case of MF66-linked DOX, an endocytic mechanism occurs, and the drug has to be released from the surface of the nanoparticle in the lysosomes. Therefore, DOX takes more time to reach the nucleus and to bind to DNA.

A detailed visualization of MF66-DOX samples showed three types of morphological alterations, compared to cells incubated with bare MF66. Mostly, aberrant metaphases, increased size of the cells and multinucleation, as evidence of mitotic catastrophe and senescence, which were confirmed by the significant increase in G2/M and polyploidy phases observed in cell cycle analysis and by positive staining for the senescence-associated β-galactosidase assay. In addition, to a lesser extent, cells with apoptotic features were detected; this process was confirmed by an increase in cells in the subG0 region in the cell cycle and by the immunofluorescence of activated caspase-3, the main effector caspase in apoptosis [25].

In summary, MF66-DOX treatment induced aberrant mitosis, apoptosis and polyploid giant senescent cells. In fact, the true roles of these cells have generated a certain amount of controversy. For many decades, senescence has been considered a conventional response to cancer therapy, taking into account that cellular senescence represents a state of cell cycle arrest in which cells remain viable and metabolically active but non-proliferative [26]. However, recent evidence has proposed that giant senescent cells, which remain chronically present following an anticancer therapy, may contribute to cancer regrowth and promote systemic inflammation, increasing the side effects of conventional anticancer agents (reviewed in [27]).

Time-lapse videomicroscopy results demonstrate that senescent giant cancer cells can undergo apoptosis. In this sense, the use of pharmacological activators of apoptosis has been proposed as an effective approach to eliminate the potential senescent giant cells generated, and thus reduce the possibilities of cancer recurrence [27,28,29].

Our results also indicate that DOX conjugated electrostatically to the MF66, once internalized, was slowly released from MF66 MNPs and was able to reach the nucleus and induce its cytotoxic effects.

On the other hand, flow cytometry results show that cells internalized the DOX attached to MF66 MNPs more slowly than free DOX, and this effect might be due to the difference between the endocytosis of the nanocarrier and the passive diffusion of free DOX [30]. However, there was higher intracellular retention of DOX when it was vehiculated in nanoparticles in comparison to free DOX. This result may be explained by the rapid exit of the internalized free DOX from the cells by overexpression of drug efflux pumps, which facilitate the acquisition of chemoresistance mechanisms [31]. This effect is blocked when DOX is loaded in different nanoplatforms [32,33]. So, the administration of electrostatic DOX conjugated to MF66 could act as an intracellular reservoir of the drug that would progressively release DOX molecules to induce their cytotoxic effect, thus bypassing the action of drug-efflux pumps.

In summary, MF66 MNPs possess appropriate properties to be considered as a suitable carrier for different agents (such as DOX) via electrostatic interactions. In fact, in a previous study, we analysed the effectiveness of MF66-DOX in combination with hyperthermia after intratumoural injection of the nanoparticles in vivo [34]. In the same study, we detected a greater cytotoxic effect on MDA-MB-231 cells of free DOX relative to MF66-DOX, possibly due to their different internalization mechanisms (passive diffusion vs. endocytosis). However, the mechanisms of action of MF66 with electrostatic formulations in MDA-MB-231 cells were not analysed in depth in the abovementioned research.

Once the high potential of the electrostatic formulations was demonstrated, our research focused on the study of MF66 nanoparticles for DOX delivery using three different covalent linkers: disulfide (DOX-S-S-Pyr, called MF66-S-S-DOX), imine (DOX-I-Mal, called MF66-I-DOX) and both disulfide and imine (DOX-I-S-S-Pyr, called MF66-S-S-I-DOX).

Our covalent strategy provides several advantages compared with the electrostatic approach, mainly due to the inherent stability of the covalent ones. For instance, these MNPs could improve the plasma circulation of the drug, preventing their non-specific release triggered by the presence of salts and proteins in the plasma, as can occur in the electrostatic approach. Furthermore, the covalent-modified systems can increase the selectivity of the release of the drug towards cancer cells, where the specific triggering stimuli are present, thus avoiding the damage in healthy tissues. In this sense, the use of disulfide moieties is particularly useful, since the release of the therapeutic molecules can be controlled by glutathione (GSH), which is present at micromolar concentrations outside the cells, but at millimolar concentrations inside them [35]. What is more, cancer cells present even higher concentrations of GSH compared to healthy cells, increasing the overall selectivity of the approach toward cancer cells [36]. Ulbrich et al. carried out a detailed review describing the most significant advantages and disadvantages related to targeted drug delivery with polymers and magnetic nanoparticles [37].

Our first results show that cell toxicity depended on the type of covalent linker used to bind DOX to the MF66 nanoparticles. Thus, MF66-S-S-I-DOX MNPs with a double-sensitive linker (to pH and redox) exhibited the greatest cytotoxic effect (only 37% viable cells). This result is difficult to compare with those obtained by other authors with superparamagnetic iron oxide nanoparticles linked covalently to DOX. As can be inferred from the aforementioned review by Ulbrich et al., different parameters and experimental protocols had been used, including among others, type of cell line, size, shape, and coating of the nanoparticle and concentration and incubation time [37].

On the other hand, similarly to the electrostatic formulation, Prussian blue staining allowed us to detect high retention rates of covalent formulations inside the cells, as well as, different alterations in cellular morphology depending on the type of covalent samples, mainly apoptosis and mitotic catastrophe. According to our results, several studies have reported an apoptotic response after incubation with DOX covalently bonded to different types of iron oxide magnetic nanoparticle [38,39]. However, there are very few studies related to mitotic catastrophe for DOX-loaded nanoparticles [40]. It is important to note that, at least to our knowledge, this is the first time that an iron oxide nanoparticle DOX loading covalently has been described to be capable of inducing cell death at the same time by both the mechanisms of apoptosis and mitotic catastrophe. One possible explanation is that in many studies with nanoparticles, neither the possible changes induced in the cell cycle profile nor inverted microscope observations of the cell response were usually evaluated, although they should be carried out to define the cell death mechanisms triggered by anticancer therapies [41].

According to all these results, the best formulation of DOX-loaded MNPs was MF66-S-S-I-DOX because drug delivery induced the highest cell death by apoptosis and the highest decrease in cell viability and, furthermore, DOX was observable in the nucleus after 72 h post-incubation. Therefore, these MF66-S-S-I-DOX MNPs were chosen to continue in subsequent studies.

In an effort to explore the in vitro properties of this covalent nanoformulation, we evaluated its effect against cancer stem cells contained in the MDA-MB-231 cell line by a mammosphere forming efficiency (MFE) assay, which is an experimental technique commonly used to analyse the efficacy of different treatments on the activity of cancer stem cells [42,43].

Several articles have stressed the potential of functionalized nanomaterials to reduce this population of cancer cells, which is responsible for relapse and metastasis [44], and our formulation presented excellent properties in this regard. In particular, MF66-S-S-I-DOX induced a decrease in the efficiency of cancer stem cells to form mammospheres compared with both the control and cells treated with bare MF66, also triggering an apoptotic response in the mammospheres formed.

Importantly, these double-sensitive MNPs were also able to induce a significant toxicity as well as a decrease in the formation of mammospheres in the primary BB3RC79 cells, obtained from a patient with a similar origin (pleural effusion) and genetic profile (triple-negative breast cancer) than MDA-MB-231 cells.

Several studies have revealed that the mammosphere-forming cells from primary breast cancer samples exhibit resistance to multiple chemotherapeutic drugs and therefore are a useful resource to test breast cancer stem-like cells targeted therapies [45,46]. In this line, our results show a remarkable effect since the patient from whom the primary cells were obtained had been treated with epirubicin (a similar drug to DOX), inducing an ineffective response in the patient who developed metastasis. By contrast, our formulation was efficient against these cells, even reducing the cancer stem cell activity.

Therefore, double-sensitive nanoparticles MF66-S-S-I-DOX have many of the properties that an ideal nanocarrier should have, such as stability, biocompatibility, sufficient loading capacity and the ability to maintain its cytotoxic activity after their internalization by tumour cells, including against cancer stem cells.

These results stimulate future studies with MF66-S-S-I-DOX-nanoformulations to determine the expression of breast CSC markers after treatments with these nanoparticles, as well as with in vivo model systems, in order to confirm their cellular functions and their therapeutic potential applications.

## 4. Materials and Methods

### 4.1. Synthesis of Nanoparticles

The MNPs used in this study, called “MF66” (Liquids Research Limited; Bangor, Gwynedd, UK), were produced by means of the co-precipitation technique [47]. Coating with dimercaptosuccinic acid (DMSA) was performed as described previously [48].

### 4.2. Synthesis of Doxorubicin Derivatives

The DOX derivatives DOX-S-S-Pyr and (5-maleimidovaleroyl) hydrazone (DOX-I-Mal) were synthesized as previously described [49,50,51]. DOX-I-S-S-Pyr was synthesized as previously described with modifications [52] (see Supplementary Material for the details on the synthesis of the different DOX derivates).

### 4.3. Electrostatic and Covalent Functionalization of MNPs

All details have been included in Supplementary Material.

### 4.4. DOX Release Studies

The release of immobilized DOX onto electrostatic formulation was monitored by a spectrophotometer. Water was used to determine the stability of the formulation over time. Then, the same experiments were performed in PBS buffer (pH 7.4), AcONa/AcOH buffer (pH 4.7) and water desorption of DOX from the MNPs in the presence of salts.

The release of DOX from covalent functionalized MF66 was carried out under extracellular pH conditions (pH 7.4 and 37 °C) using two concentrations of reducing agent (1 μM and 1 mM of 1,4-dithiothreitol (DTT), mimicking the extracellular and intracellular reducing power, respectively. Intracellular pH conditions (pH 4.7 and 37 °C) were carried out using two concentrations of reducing agent (1 μM and 1 mM of DTT). More details can be found in Supplementary Material.

### 4.5. Cell Culture

The human breast cancer cell line MDA-MB-231 was obtained from American Type Culture Collection (ATCC^®^ HTB-26TM) and was cultured following the standard protocols for established cell lines [53]. Primary cells BB3RC79 derived from a patient with triple negative breast cancer were cultured with DMEM/F12 medium (DMEM/Ham’s F12; Thermo Fisher Scientific), supplemented with 10% FBS, 2 mM L-glutamine (Thermo Fisher Scientific), 50 U mL^−1^ penicillin and 50 μg mL^−1^ streptomycin and 10 μg mL^−1^ insulin (Merck), 1 μg mL^−1^ hydrocortisone (Merck) and 50 ng mL^−1^ epidermal growth factor receptor (EGFR) (Miltenyi Biotec; Bergisch Gladbach, Germany). Metastatic samples from breast cancer patients were collected at The Christie NHS Foundation Trust. Patients were informed and consented according to local National Research Ethics Service guidelines (Ethical Approval Study No.: 05/Q1402/25).

### 4.6. Nanoparticles Preparation

The different MNP stocks at 2.4 mg Fe mL^−1^ were dispersed by sonication for 5 min in a 50 kHz sonicator bath (Bath Ultrasonic QS3, Scientific Laboratory Supplies; Cardiff, UK). MNPs were then resuspended in complete cell culture media at a final concentration of 0.1 mg Fe mL^−1^ (and drugs immobilized were at 4 µM for DOX in the electrostatic formulations and 2 µM for DOX in the covalent formulations). The mixture was sonicated for 1 min and incubated with cells for 24 h. The studies were evaluated at different post-incubation times (mainly, from 0 to 72 h).

### 4.7. Cytotoxicity Analysis

Cell viability with the different formulations was assessed by AlamarBlue^®^ assay in MDA-MB-231 cells and trypan blue in BB3RC79 cells according to manufacturer instructions (for more details see Supplementary Material).

### 4.8. Nanoparticles Internalization and Cancer Cell Morphology

These parameters were analysed in living cells to avoid artefacts of fixation and in cells fixed and stained with Prussian blue specific to visualize iron nanoparticles [54].

### 4.9. Analysis of Internalized DOX into Cells

MDA-MB-231 cells seeded on 25 cm^2^ flasks were incubated with the electrostatic MF66-DOX formulation or ‘free DOX’ (non-covalently bounded DOX) for 24 h with different post-incubation times: 0, 24 and 72 h. Then, the supernatant was removed (harvesting detached cells) and cells attached to well, were trypsinized (Thermo Fisher Scientific). These cells were added to the previously collected ones and centrifuged for 4 min at 300 g (JP Select; Abrera, Spain). The supernatant was discarded, and the pellet was resuspended in cold medium without phenol red (Thermo Fisher Scientific). Samples were analysed in a flow cytometer Cytomics FC500 (Beckman-Coulter; Brea, CA, USA) with a laser of 488 nm and a filter 620 BP.

### 4.10. Analysis of Cellular Inactivation Mechanisms

#### 4.10.1. Cell Cycle Analysis

MDA-MB-231 cells, grown directly onto wells, were incubated with the different samples for 24 h, washed, collected, and analysed after 72 h. To this purpose, we added 50 μL of RNAse 4 kU mL^−1^ and 1 mL of propidium iodide 50 µg mL^−1^ of kit DNA-Prep Reagents (6607055, Beckman-Coulter). Samples were stirred and incubated for 30 min at 37 °C in darkness. They were analysed in a flow cytometer Cytomics FC500 with a laser of 488 nm and a filter 620 BP.

#### 4.10.2. Indirect Immunofluorescence for Cleaved Caspase-3 and Cytochrome c

The common protocol for indirect immunofluorescence was performed (see more details in Supplementary Material) [55].

#### 4.10.3. Cellular Senescence Assessment

MDA-MB-231 cells were seeded on coverslips, incubated with the different formulations for 24 h and analysed 96 h after with senescence β-galactosidase staining kit (CS0030, Merck) following the instructions of the manufacturer.

#### 4.10.4. Videomicroscopy

Cells seeded in chambered coverslips (Ibidi; Martinsried, Germany) were treated as previously explained. Frames were acquired by phase contrast microscopy every 15 min between 48 and 72 h after incubation, maintaining CO_2_, temperature and humidity conditions in cell culture range.

### 4.11. Mammosphere Forming Efficiency

Breast stem cell activity was quantified by standard sphere-forming assay (see Supplementary Material) [42]. Mammospheres bigger than 50 μm diameters were counted after 5 or 7 days for MDA-MB-231 or BB3RC79 cells, respectively.

### 4.12. Mammosphere Morphology and Analysis

Mammospheres fixed in formalin were embedded in paraffin. Sections of 5 µm were deparaffinised and analysed by the different protocols (see Supplementary Material).

### 4.13. Statistical Analysis

Results are the mean values and standard deviation (SD) from at least five different experiments in triplicate. Statistical analysis was performed by GraphPad Prism 5 software (GraphPad Inc.; La Jolla, CA, USA) using one-way ANOVA and Tukey’s post-test. The threshold for significance was *p* = 0.05 and statistically significant differences were labelled as ‘*’ when *p* < 0.05, ‘**’ when *p* < 0.01 and ‘***’ when *p* < 0.001.

## 5. Conclusions

In summary, this study brings essential insights into the relevance of the selection of appropriate functionalization strategies, which have significant implications on the final performance of a nanoformulation. Among others, the drug release mechanism and kinetics can be achieved, leading to different cytotoxic efficacy and cell death mechanisms. The best performing functionalized nanoparticle in this study (MF66-S-S-I-DOX) is a promising tool, which can be used to improve the efficiency of existing chemotherapeutic approaches with iron oxide nanoparticles, reducing the side effects of the chemotherapeutic drug and increasing efficiency against cancer stem cells.

## Supplementary Materials

The following are available online at https://www.mdpi.com/2072-6694/12/6/1397/s1. Supplementary Materials: 1.1. Electrostatic functionalization of MNPs, 1.2. Covalent functionalization of MNPs, 1.3. DOX release studies, 1.4. AlamarBlue® assay, 1.5. Trypan blue assay, 1.6. Indirect immunofluorescence for cleaved caspase-3 and cytochrome c, 1.7. Mammosphere forming efficiency, 1.8. Morphology of mammospheres, 1.9. Statistical analysis, Supplementary Results: 2.1. Morphological effect of electrostatic formulations over time, Supplementary Movie S1: Videomicroscopic analysis of control MDA-MB-231 cells, Supplementary Movie S2: Videomicroscopy study of MDA-MB-231 cells incubated with MF66, Supplementary Movie S3: Videomicroscopy study of MDA-MB-231 cells incubated with MF66-DOX, 2.2. Internalization and morphological alterations of covalent formulations in living cells, Table S1: Characterization of the DOX functionalized MF66-MNP, Figure S1: Surviving fraction of MDA-MB-231 cells incubated 24 h with free unmodified DOX, Figure S2: Living cells visualized 72 h after incubation for 24 h with the different formulations linked covalently to DOX.
