# Supplementary material for: Tailored Functionalized Magnetic Nanoparticles to Target Breast Cancer Cells Including Cancer Stem-Like Cells
Source: Cancers (Basel). 2020 May 29;12(6):1397. doi: 10.3390/cancers12061397 (PMC7352336; doi:10.3390/cancers12061397)
Supplement: Supplementary file 1 [file cancers-12-01397-s001.zip › suppl/SUPPLEMENTARY_MATERIAL_AV.doc]

Electronic Supplementary Information

Tailored functionalized magnetic nanoparticles to target breast cancer cells including cancer stem-like cells

Ana Lazaro-Carrillo, Macarena Calero, Antonio Aires, Aitziber L. Cortajarena, Bruno M. Simões, Alfonso Latorre, Álvaro Somoza, Robert B. Clarke, Rodolfo Miranda, Angeles Villanueva*

**1. METHODS**

**1.1. Synthesis of doxorubicin derivatives**

The structures of the resulted DOX derivatives are presented in the scheme S1.

**Scheme S1.** Structure of DOX derivatives: DOX-S-S-Pyr (MF66-S-S-DOX), DOX-I-Mal (MF66-I-DOX) and DOX-I-S-S-Pyr (MF66-S-S-I-DOX).

**1.1.1. Synthesis of DOX-S-S-Pyr (see Reference** [1]**).**

**Scheme S2.** Synthesis route of DOX-S-S-Pyr (MF66-S-S-DOX).

**Synthesis of 2-(Pyridin-2-yldisulfanyl)ethanol** **(1)**

To a solution of aldrithiol (300 mg, 1.36 mmol) in MeOH (1.5 mL) under Ar, 2-mercaptoethanol (53 μL, 0.75 mmol) was added slowly and stirred for 16 h. Then, the solvent was evaporated in vacuum and the residue purified by flash chromatography (CH_2_Cl_2_/AcOEt 5:1) to obtain compound **1** as a colorless oil in 86% yield; ^1^H NMR (300 MHz, CDCl_3_) δ 8.51 (d, *J* = 4.3 Hz, 1H), 7.58 (td, *J* = 8.0, 1.7 Hz, 1H), 7.40 (d, *J* = 8.0 Hz, 1H), 7.17 – 7.13 (m, 1H), 5.72 (bs, 1H), 3.80 (dd, *J* = 10.4, 6.5 Hz, 2H), 2.97 – 2.94 (m, 2H).

**Synthesis of 4-Nitrophenyl 2-(pyridin-2-yldisulfanyl)ethyl carbonate (2)**

To a solution of compound **1** (100 mg, 0.53 mmol), and bis(4-nitrophenyl) carbonate (241 mg, 0.79 mmol) in CH_2_Cl_2_ (2 mL) under Ar, DIPEA (158 μL, 0.79 mmol) was added and stirred for 5 h. The mixture was washed with water, and the organic phase dried with MgSO_4_. After solvent evaporation, the residue was purified by flash chromatography (Hexane/AcOEt 4:1 and then 2:1) to obtain compound **2** as a colorless oil 67% yield; ^1^H NMR (300 MHz, CDCl_3_) δ 8.50 (d, *J* = 4.8 Hz, 1H), 8.28 (d, *J* = 9.1 Hz, 2H), 7.72 – 7.59 (m, 2H), 7.38 (d, *J* = 9.1 Hz, 2H), 7.15 – 7.10 (m, 1H), 4.57 (t, *J* = 6.4 Hz, 2H), 3.16 (t, *J* = 6.4 Hz, 2H).

**Synthesis of Doxorubicin derivative, DOX-S-S-Pyr**

To a solution of compound **2** (10 mg, 0.028 mmol) and doxorubicin hydrochloride (12 mg, 0.020 mmol) in DMF (1 mL) under N_2_, DIPEA (8 µL, 0.028 mmol) was added at room temperature and stirred for 16 h. Then, the solvent was evaporated and the residue was purified by flash chromatography (eluent: CH_2_Cl_2_/MeOH 20:1) to obtain compound **3** as red solid in 90% yield; ^1^H NMR (500 MHz, CDCl_3_) δ 13.94 (s, 1H), 13.18 (s, 1H), 8.40 (d, *J* = 3.8 Hz, 1H), 8.00 (d, *J* = 7.6 Hz, 1H), 7.76 (t, *J* = 8.0 Hz, 1H), 7.70 (d, *J* = 7.5 Hz, 1H), 7.63 (t, *J* = 7.4 Hz, 1H), 7.37 (d, *J* = 8.5 Hz, 1H), 7.07 (t, *J* = 1H), 5.50 b(s, 1H), 5.26 (dd, *J* = 3.7, 2.0 Hz, 1H), 5.15 (d, *J* = 8.8 Hz, 1H), 4.74 b(s, *J* = 21.4 Hz, 2H), 4.56 (bs, 1H), 4.42 – 4.34 (m, 1H), 4.21 – 4.08 (m, 2H), 4.06 (s, 3H), 3.81 (m, 1H), 3.62 (bs, 1H), 3.22 (dd, *J* = 18.8, 1.5 Hz, 1H), 3.14 – 2.86 (m, 5H), 2.33 (d, *J* = 14.6 Hz, 1H), 2.15 (dd, *J* = 15.0, 3.6 Hz, 1H), 1.81 (m, 2H), 1.48 – 1.42 (m, 2H), 1.29 (d, *J* = 6.5 Hz, 3H); ^13^C NMR (126 MHz, CDCl_3_) δ 213.9, 187.0, 186.6, 161.0, 160.1, 156.2, 155.6, 155.2, 149.8, 149.4, 137.3, 135.7, 135.4, 133.6, 133.6, 120.9, 120.8, 119.9, 119.8, 118.4, 111.5, 111.4, 100.9, 69.7, 69.0, 67.4, 65.6, 63.5, 56.7, 53.4, 47.0, 37.5, 35.6, 33.9, 30.0, 29.7, 28.3, 17.0; MS (ESI): m/z (%) 757(100).

**1.1.2. Synthesis of DOX-I-S-S-Pyr (see Reference** [2]**).**

**Scheme S3.** Synthesis route of DOX-I-S-S-Pyr (MF66-S-S-I-DOX).

To a solution of **2** (55 mg, 0.15 mmol) in CH_2_Cl_2_ (0.7 mL) under Ar, hydrazine (15 μL, 0.47 mmol) and DIPEA (27 μL, 0.15 mmol) were added at 0 ◦C. The reaction was warmed to room temperature and stirred for 3 h. After concentration in vacuo, the residue was purified by flash chromatography using CH_2_Cl_2_ and MeOH (5%) as eluent to give 8 mg (0.03 mmol) of the corresponding carbazate intermediate, which was reacted in MeOH (0.45 mL) with DOX.HCl (6 mg, 0.01 mmol) in the presence of two drops of TFA, at room temperature during 16 h. The reaction was concentrated in vacuo, and the residue washed with acetonitrile to give DOX-I-S-S-Pyr. ^1^H NMR (500 MHz, MeOD) δ 7.96 – 7.70 (s, *J* = 22.2 Hz, 1H), 7.86 – 7.70 (m, 3H), 7.55 (d, *J* = 7.7 Hz, 1H), 7.21 (s, 1H), 5.51 (s, 1H), 5.18 (s, 1H), 4.63 (dd, *J* = 24.4, 14.8 Hz, 2H), 4.41 (s, 2H), 4.27 (dd, *J* = 13.7, 7.2 Hz, 1H), 4.03 (s, *J* = 9.0 Hz, 3H), 3.67 (s, 1H), 3.56 (dd, *J* = 9.0, 3.6 Hz, 1H), 3.13 (t, *J* = 6.0 Hz, 2H), 3.10 – 2.99 (m, 1H), 2.47 – 2.33 (m, 2H), 2.04 (td, *J* = 12.8, 3.7 Hz, 1H), 1.91 (dd, *J* = 12.7, 3.8 Hz, 1H), 1.32 (d, *J* = 6.5 Hz, 4H). MS (ESI): m/z (%) 771.1937 (M+H, 100).

**1.1.3. Synthesis of DOX-I-Mal (see Reference** [3]**).**

**Scheme S4.** Synthesis route of DOX-I-Mal (MF66-I-DOX).

**Sinthesis of 2,5-dioxopyrrolidin-1-yl 5-(2,5-dioxo-2,5-dihydro-1H-pyrrol-1-yl)pentanoate (3).**

To a solution of maleic anhydride (167mg, 1.7 mmol) in DMF (2 mL), aminovaleric acid (200 mg, 1.7 mmol) was added in one portion and stirred for 3 h. Then, the solution was cooled to 0 ºC and NHS (234 mg, 2 mmol) and DCC (700 mg, 3.4 mmol) were added successively. The reaction was warmed to room temperature and stirred 16 h. The solid was filtered off, and the filtrate concentrated in vacuo and purified by flash chromatography using CH_2_Cl_2_/IPA (2%) as eluents to give **3** in 50% yield. ^1^H NMR (300 MHz, CDCl_3_) δ 6.68 (s, 2H), 3.56 (t, *J* = 5.7 Hz, 2H), 2.82 (s, 4H), 2.64 (t, *J* = 6.2 Hz, 2H), 1.76 – 1.66 (m, 4H).

**Synthesis of DOX-I-Mal**

To a solution of **3** (50 mg, 0.17 mmol) in CH_2_Cl_2_ (1 mL) under Ar and at 0 ºC, BOCNHNH_2_ was added and stirred for 3 h. The reaction was concentrated in vacuo and purified by flash chromatography using CH_2_Cl_2_/MeOH (5%) as eluent to give the corresponding Boc protected carbazate., which was treated with TFA (0.5 mL) at 0 ºC during 10 min, and concentrated in vacuo to obtain 15 mg (0.05 mmol) of carbazate intermediate. This compound was dissolved in MeOH (4.5 mL) and treated with DOX.HCl (13 mg, 0.023 mmol) during 16 h in the presence of 1 drop of TFA. The reaction was concentrated in vacuo, and the residue dissolved in the minimum quantity of MeOH. DOX-I-Mal was obtained after precipitation with acetonitrile. ^1^H NMR (300 MHz, MeOD) δ 7.96 – 7.74 (m, 2H), 7.54 (d, *J* = 7.5 Hz, 1H), 6.79 (s, 2H), 5.48 (s, 1H), 5.08 (s, 1H), 4.75 – 4.59 (m, 1 H), 4.64 (d, *J*= 3.7 Hz, 1H), 4.33 – 4.15 (m, 1H), 4.02 (s, 3H), 3.70 (s, 2H), 3.64 – 3.50 (m, 2H), 3.41 (s, 2H), 3.07 (s, 1H), 2.89 (d, *J* = 17.8 Hz, 1H), 2.41 (m, 4H), 2.16 – 1.83 (m, 2H), 1.68 – 1.47 (m, 4H), 1.32 (t, *J* = 6.5 Hz, 3H). MS (ESI): m/z (%) 737.2638 (M+H, 100).

**1.2. Electrostatic functionalization of MNP**

MF66 were dispersed by sonication for 5 min in a 40 kHz sonicator bath (Branson 3510 ultrasonic cleaner, Thomas Scientific, Swedesboro, USA). Then these stocks and DOX solution were sterilized by 0.22 µm pore size filtration (Merck; Darmstadt, Germany).

For the electrostatic immobilization, 202 µl of a DOX hydrochloride solution (500 µM, Fluorochem) were incubated with 1 mL MF66 for 6 h at room temperature in a rocking shaker. MF66-DOX was centrifuged and the supernatant was removed. MNP pellets were redispersed at 2.4 mg Fe mL^-1^.

Immobilized DOX was quantified by measuring the fluorescence of unbound DOX in the supernatant (λ_exc_ = 495 nm, λ_em_ = 595 nm).

**1.3. Covalent functionalization of MNP**

1.3.1. Pre-activation of MNP

MF66 were first modified with cysteamine hydrochloride to introduce thiol moieties (thiolated MF66). To 1 mL of MF66 at 2.4 mg Fe mL^-1^ were added 50 μmol of cysteamine hydrochloride g^-1^ Fe, previously neutralized by 1 equivalent of NaOH, 150 μmol of EDC g^-1^ Fe and 75 μmol of NHS g^-1^ Fe. After 16 h, the sample was washed by cycles of centrifugation and redispersion in PBS buffer (pH 7.4) at least 3 times.

1.3.2. Reaction between thiolated MF66 and DOX derivatives (DOX-S-S-Pyr, DOX-I-Mal and DOX-I-S-S-Pyr)

1 mL of an aqueous suspension of thiolated MF66 at 2.4 mg Fe mL^-1^ was mixed with 40 μmol of DOX derivatives g^-1^ Fe (14.4 μL of DOX derivatives solution at 5 mM in DMF) during 16 h at 37 °C. After incubation, 20 mL of brine were added and the sample centrifuged 10 min at 5000 g. From the collected supernatants, the covalently immobilized DOX onto thiolated MF66 was determined by measuring the absorbance of the sample at 495 nm with UV-vis spectrophotometer as the difference between the amount of unbound DOX derivative in the supernatant after the immobilization and the total DOX derivative used for the immobilization (μmol DOX mg^-1^ Fe). Finally, the sample was redispersed in 1 mL of PBS buffer (pH 7.4).

**1.4. DOX release studies**

1.4.1. DOX release from electrostatic functionalized MF66-MNP

As previously mentioned, the release of immobilized DOX onto MF66 was monitored in water to determine the stability over time and in PBS buffer (pH 7.4) or AcONa/AcOH buffer (pH 4.7). Functionalized MF66 nanoparticles were dispersed at a final concentration of 0.3 mg Fe mL^-1^. The samples were then placed at 37 °C and at different time points (up to 120 h), 100 μl of each sample were collected, centrifuged and the supernatants were analyzed. The amount of DOX released was analyzed by measuring the absorbance of the sample at 495 nm with a UV-vis spectrophotometer at regular time intervals. The percentage of drug released was calculated from a standard calibration curve of free drug solution.

1.4.2. DOX release from covalent functionalized MNP

The cumulative drug release experiments were carried out using four different conditions to evaluate the stimuli response behaviour of functionalized MF66 toward reducing and pH environment, as we mentioned. For each experiment, 2.4 mg of DOX functionalized MF66-MNP were resuspended in 1 mL of 10 mM phosphate buffer at pH 7.4 containing 1 μM of DTT or 1 mM DTT, or in 1 mL of 10 mM sodium acetate buffer at pH 4.7 containing 1 μM of DTT or 1 mM DTT, and incubated at 37 °C. The amount of DOX released was analyzed by measuring the absorbance of the sample at 495 nm with a UV-vis spectrophotometer at regular time intervals. The percentage of drug released was calculated from a standard calibration curve of free drug solution.

**1.5. AlamarBlue^®^ assay**

MDA-MB-231 cells seeded in 96-multiwell plates were incubated with different samples for 24 h at the concentration indicated above. Samples were washed and 100 μl of AlamarBlue^®^ (Thermo Fisher Scientific) were added into each well at a concentration of 100 µl mL^-1^ in complete medium. Cells were maintained in the incubator at 37 °C for 3 or 4 h. After this time, the liquid of each well was transferred to a black 96-multiwell plate and fluorescent emission was measured at 590 nm (λ excitation 530 nm) on a plate reader Synergy HT (BioTek; VT, USA). This protocol was repeated at different post-incubation times (between 0 and 72 h). Survival fraction was expressed as the percentage of fluorescence of treated cells respect to fluorescence of control cells (not incubated with any formulation).

**1.6. Trypan blue assay**

Cell viability in BB3RC79 cells was quantified by Trypan blue dye exclusion method. Briefly, cells were incubated 24 h with MF66 or MF66-S-S-I-DOX and 24 h after, trypsin was added to control and treated cells. After cells were detached from the plate, they were resuspended in culture media. Equal volumes of each cell suspension and trypan blue solution (0.2% in PBS) were mixed and used for cell counting by hemocytometer. Blue-stained cells were counted as non-viable cells and unstained cells as viable cells.

**1.7. Indirect immunofluorescence for cleaved caspase-3 and cytochrome c**

For indirect immunofluorescence detection of active caspase-3, MDA-MB-231 cells on coverslips were fixed in formol-PBS 1:10 (Merck) for 20 min at 4 °C, washed three times with PBS (5 min each) and permeabilized with 0.5% Triton X-100 (Merck) at 0.5% in PBS. After 5 min, Triton X-100 was removed and cells were incubated in blocking solution (5% bovine serum albumin, 5% FBS, 0.02% Triton X-100 in PBS) for 30 min at room temperature. Once removed from the blocking solution, 1:100 solution of primary monoclonal rabbit anti-cleaved caspase-3 antibody (9664S, Cell Signaling Technology; Danvers, MA, USA) was added to each sample and incubated at 37 °C for 1 h. Three 5-min washings with PBS were then carried out before addition of Triton X-100 for 5 min. Incubation of 1:300 solution of secondary antibody goat anti-rabbit IgG bounded to Alexa Fluor^®^ 555 (A21428, Thermo Fisher Scientific) was performed and then cells were washed. After counterstaining using Hoechst 33258 (5 μl mL^-1^) for 3 min, the samples were mounted with ProLong^®^ Gold reagent (Thermo Fisher Scientific).

In cytochrome c case, similar protocol was used but monoclonal mouse anti-cytochrome c antibody (33-8200, Thermo Fisher Scientific) (dilution 1:25) was used as primary antibody and goat anti-mouse IgG bounded to Alexa Fluor® 488 (dilution 1:100) as secondary antibody.

**1.8. Mammosphere forming efficiency**

Cells were trypsinized and centrifuged at 400 g for 2 min 72 h after 24 h treatment in MDA-MB-231 cells and 24 h after in primary cells. The supernatant was removed and the pellet was resuspended in 1–5 mL of ice-cold PBS. This solution was passed through a 25-gauge needle (Terumo Corporation; Tokyo, Japan) using a 1-mL syringes (BD; Franklin Lakes, NJ, USA) 3-5 times to disperse cells into single cell suspension. Cells were seeded at 500 cells cm^-2^ in mammosphere media [phenol red-free DMEM/12 (Thermo Fisher Scientific) containing 2% B-27^®^ supplement minus retinoic acid (Thermo Fisher Scientific) and 20 ng mL^-1^ recombinant epidermal growth factor (Merck)] in non-adherent plates (poly-HEMA; Merck).

Al previously mentioned, mammospheres bigger than 50 μm diameter were counted after 5 or 7 days for MDA-MB-231 or BB3RC79 cells respectively. Mammosphere forming efficiency (%) was calculated as follows: Number of mammospheres per well / Number of cells seeded per well x 100.

**1.9. Morphology of mammospheres**

Mammospheres were collected and centrifuged at 120 g for 2 min at RT, and the supernatant was then discarded. Samples were fixed in formalin (Merck) and centrifuged again. Finally, the pellet was resuspended in 100 μl PBS and embedded in paraffin. Sections of 5 µm were deparaffinised before each staining using the following protocol: xylene 15 min, 100% ethanol 10 min twice, 96% ethanol 5 min, 70% ethanol 5 min and distilled water 5 min.

*Prussian blue staining.* Sections were stained with an equal volume of 1 N hydrochloric acid and 10% potassium ferrocyanide trihydrate for 15 min, and counterstained with 1% neutral red for 2 min.

*Indirect immunofluorescence for cleaved caspase-3 and cytochrome c*. Immunofluorescences were performed with an identical protocol to the above mentioned.

**References:**

(1) Latorre, A.; Couleaud, P.; Aires, A.; Cortajarena, A. L.; Somoza, Á. Multifunctionalization of Magnetic Nanoparticles for Controlled Drug Release: A General Approach. Eur. J. Med. Chem. 2014, 82, 355–362**.**

(2) Kaneko, T.; Willner, D.; Monkovic, I.; Knipe, J. O.; Braslawsky, G. R.; Greenfield, R. S.; Vyas, D. M. New Hydrazone Derivatives of Adriamycin and Their Immunoconjugates - a Correlation between Acid Stability and Cytotoxicity. Bioconjug. Chem. 1991, 2 (3), 133–141.

**(**3) Furgeson, D. Y.; Dreher, M. R.; Chilkoti, A. Structural Optimization of a “Smart” Doxorubicin–Polypeptide Conjugate for Thermally Targeted Delivery to Solid Tumors. J. Control. Release 2006, 110 (2), 362–369.

**2. RESULTS**

**2.1. Functionalization of MF66-MNP**

**2.1.1. Electrostatic functionalization of MF66-MNP**

As a first approach to improve cancer therapy, we have used the non-covalent functionalization of nanoparticles, mainly through electrostatic or hydrophobic interactions. This approach produced stable MF66 loaded with 40 μmol DOX g^-1^ Fe (95% of immobilization yield). Zeta potential and hydrodynamic diameter obtained for this formulation are showed in Table S1.

**2.1.2. Covalent functionalization of MF66**

**2.1.2.1. Pre-activation of MF66 by cysteamine**

The introduction of thiol groups on the surface of MF66 was achieved by reaction between cysteamine hydrochloride and carboxylate groups of MF66 in presence of EDC/NHS in basic medium. Particularly, the reaction of 50 μmol of cysteamine g^-1^ Fe led to a maximum of 30 μmol of reactive thiol groups g^-1^ Fe.

**2.1.2.2. Covalent attachment of Doxorubicin on pre-activated MF66**

The reaction of the DOX derivatives with pre-activated MF66 led to stable colloidal formulations of functionalized MF66 with 20 μmol DOX g^-1^ Fe in all the cases. The immobilization yields were around 50% for DOX. Zeta potentials and hydrodynamic diameters obtained for each formulation are showed in Table S1.

**Table S1**. Characterization of the DOX functionalized MF66-MNP

| Sample | Zeta potential (mV) | Hydrodynamic diameter (nm) | PDI |
| --- | --- | --- | --- |
| MF66 | -56.8 | 94.8 | 0.18 |
| MF66-DOX | -41.9 | 119.3 | 0.16 |
| MF66-S-S-DOX | -46.7 | 95.3 | 0.19 |
| MF66-I-DOX | -46.5 | 96.8 | 0.16 |
| MF66-S-S-I-DOX | -42.7 | 96.4 | 0.20 |

**2.2 Cytotoxicity of free doxorubicin (DOX)**

MDA-MB-231 cells were incubated with free DOX at 4 μM (the equivalent molar amount of MF66-DOX in the electrostatic formulations) for 24 h and cellular viability assessed by AlamarBlue® assay 72 h after incubation.

**Figure S1**. Surviving fraction of MDA-MB-231 cells incubated 24 h with free unmodified DOX.

**2.2. Morphological effect of electrostatic formulations over time by time-lapse videomicroscopy**

The different morphological effects induced by the formulation with DOX, were corroborated by time-lapse videomicroscopy. Representative live cell imaging video of MDA-MB-231 untreated (control) cells shows the high mobility of these cell line (file Supplementary Movie S1). Recorded cells incubated with bare MF66 indicated a similar cell size, division rate and motility that control cells (file Supplementary Movie S2). By contrast, cells incubated with MF66-DOX showing flatten cells with a larger size, longer but less frequent mitosis and cell death by apoptosis. The video also shows an example of a senescent multinucleated giant cell (bottom-right corner) that finally undergoes apoptosis (file Supplementary Movie S3).

**Supplementary Movie S1.** Videomicroscopic analysis of control MDA-MB-231 cells

**Supplementary Movie S2.** Videomicroscopy study of MDA-MB-231 cells incubated with MF66.

**Supplementary Movie S3.** Videomicroscopy study of MDA-MB-231 cells incubated with MF66-DOX.

**2.3. Internalization and morphological alterations of covalent formulations in living cells**

In order to avoid artefacts by fixation, internalization of covalent formulations into the cells and pursued morphologies were analysed after 72 h post-incubation directly by microscopy.

The control cells and the cells incubated with bare MNP did not show any red fluorescence signal (not shown). However, cytoplasm in all the three covalent formulations of DOX-loaded MF66, intense red dots were mainly visualized in cytoplasm, which correspond to DOX attached to MNP. In addition, a diffuse red signal from released DOX was also visualized intracellularly in the nucleus and the cytoplasm, showing a slightly higher signal in nuclei with MF66-S-S-I-DOX. In bright field microscopy MNP appeared as brown spots of different size in cell cytoplasm, but always outside the nucleus (Figure S2 a).

The toxic effects induced by the incubation with the different covalent samples of DOX-loaded MF66 were evaluated analyzing treated living cells at 72 h post-incubation (Figure S2 b). In this way, it was observed that the cellular density gradually increased by successive cell divisions in the control cells. By contrast, a decrease of cell density cultures was detected with DOX-loaded MF66 samples. The highest decrease of cell density is shown in MF66-S-S-I-DOX sample. Moreover, a significantly larger size was visualized in treated cells in comparison to control cells. These cells could be from two to five times bigger than control cells.

**Figure S2.** Living cells visualized 72 h after incubation with the different formulations linked covalently to DOX. (**a**) Intracellular accumulation of DOX in cells incubated with MF66-S-S-DOX, MF66-I-DOX and MF66-S-S-I-DOX MNP visualized by fluorescence and bright field microscopy, respectively. Scale bar: 25 μm. (**b**) Cell morphology of control cells or incubated with MF66-S-S-DOX, MF66-I-DOX and MF66-S-S-I-DOX MNP visualized by DIC microscopy. Scale bar: 50 μm.
